# Association Between Injury Mechanisms, Hospital Admission, and Length of Stay Among Trauma Patients Aged ≥14 Years in an Adult Emergency Department in Riyadh, Saudi Arabia

**DOI:** 10.7759/cureus.117679

**Published:** 2026-10-09

**Authors:** Abdulaziz Alrabiah, Fatima M Halawi, Shatha M Alshabani, Lina F Alyahya, Abdulaziz S Almanea, Bandar M Almutairi, Lujain A Alkhalaf, Lama Al-Eyadhy

**Affiliations:** 1 Emergency Department, King Saud University, Riyadh, SAU; 2 College of Medicine, King Saud University, Riyadh, SAU

**Keywords:** emergency department, hospital admission, injury mechanisms, length of stay, road traffic accidents, trauma

## Abstract

Background: Trauma presentations to emergency departments (EDs) encompass a wide range of injury mechanisms and clinical outcomes. Mechanisms of injury may influence both hospital admission and length of stay (LOS). This study examined the association between injury mechanisms and hospital admission as the primary outcome and evaluated LOS as a secondary and exploratory outcome among trauma patients presenting to the ED in Riyadh, Saudi Arabia.

Methods: A retrospective cohort study was conducted at King Khalid University Hospital, including patients aged 14 years and older who presented with traumatic injuries between January 2019 and April 2025. Hospital admission following the initial ED evaluation was the primary outcome, while LOS among admitted patients was considered a secondary and exploratory outcome. From a database of 10,722 trauma-related records available for screening, patients were randomly selected and assessed against predefined eligibility criteria, with ineligible records replaced by additional randomly selected records until a final sample of 218 eligible ED encounters was obtained. Data were extracted from electronic medical records and analyzed using descriptive and inferential statistics. A pre-specified multivariable logistic regression model was used to assess factors associated with hospital admission.

Results: A total of 218 patients were included, with a median age of 29.5 years (IQR 22-39); 66.5% were male. Falls were the most frequently documented injury mechanism (49.1%), followed by traffic-related incidents (22.0%). Overall, 32 patients (14.7%) required hospital admission. Among the 30 admitted patients included in the LOS analysis, the median LOS was 5 days (IQR 2-14). Traffic-related injury was associated with higher odds of hospital admission after adjustment for age, sex, and any comorbidity (adjusted OR 3.41, 95% CI 1.49-7.83; p=0.004), whereas age, sex, and the presence of any comorbidity were not significantly associated with admission. Poisoning was associated with longer LOS (median 15 vs 4 days; p=0.042), although this finding was based on only three admitted patients with poisoning.

Conclusions: Falls were the most frequently documented injury mechanism in this single-center ED cohort, whereas traffic-related injuries were independently associated with higher odds of hospital admission. Poisoning was associated with longer LOS, although this finding was based on a small number of cases and should be interpreted cautiously. These findings highlight the distinction between injury mechanisms contributing to ED presentation volume and those associated with greater inpatient utilization.

## Introduction

Trauma remains a major public health concern worldwide and is associated with significant morbidity, mortality, and healthcare burden. Prolonged hospital stay, defined as hospitalization exceeding the expected duration for a specific condition or procedure, is associated with increased risks of hospital-acquired complications, including infections, pressure ulcers, and thromboembolic events. In addition, extended hospitalization places substantial pressure on healthcare resources and increases financial costs for healthcare systems [1]. In Saudi Arabia, trauma is a leading cause of death and disability, accounting for 14.4% of all fatalities in 2016, while approximately 300 injured patients were transported daily by the Saudi Red Crescent Authority in 2018 [2]. Common injury mechanisms associated with prolonged hospitalization include falls, motor vehicle accidents (MVAs), penetrating injuries, and burns [3,4].

Previous studies have shown that demographic and socioeconomic factors may influence both injury patterns and hospital length of stay (LOS). Age and gender affect the type and severity of injuries sustained, with falls being more common among older adults and trauma related to high-risk activities more frequently affecting younger individuals. Socioeconomic status has also been associated with disparities in healthcare access and recovery outcomes, potentially contributing to prolonged hospitalization [5]. Regional studies from Iran demonstrated that male patients experienced longer LOS and higher hospitalization costs, while patients with higher educational levels had shorter hospital stays [6]. Similarly, studies from the United States reported variations in LOS according to age, sex, and insurance status [7].

In Saudi Arabia, road traffic crashes (RTCs) remain the predominant mechanism of trauma. A retrospective study conducted at King Abdulaziz Medical City reported that RTCs accounted for 42.1% of blunt trauma cases, followed by penetrating injuries and burns [8]. Earlier local data also identified falls and pedestrian injuries as major contributors to trauma admissions, with a mean hospital stay of 19 days [9]. In contrast, studies from high-income countries found falls to be the leading trauma mechanism and reported shorter average LOS [10]. Comorbidities and pre-existing medical conditions further contribute to prolonged hospitalization and poorer recovery outcomes following trauma [3,11-14].

Although previous trauma-registry studies have characterized the epidemiology of injury mechanisms and their relationship with hospitalization, registry-based data may disproportionately represent patients with more severe injuries or those transferred to specialized trauma centers. An emergency department (ED)-based cohort captures a broader spectrum of trauma presentations, including injuries that are evaluated and discharged without admission, and therefore provides an opportunity to examine the relationship between injury frequency at the ED level and subsequent inpatient utilization. This distinction is clinically relevant because a mechanism that accounts for a large proportion of ED presentations may not necessarily be the mechanism associated with the greatest need for hospitalization. However, this relationship has not been well characterized in an ED-based trauma population in Riyadh. Understanding this distinction may help inform ED preparedness, inpatient resource allocation, and trauma-prevention priorities.

Therefore, this study aimed primarily to examine the association between injury mechanisms and hospital admission among trauma patients presenting to the ED in Riyadh, Saudi Arabia. As a secondary and exploratory objective, the study assessed the association between injury mechanisms and hospital LOS among admitted patients, while also examining the associations of demographic characteristics and comorbidity status with these outcomes.

## Materials and methods

Study design and setting

A retrospective cohort study was conducted at King Khalid University Hospital (KKUH). Data were retrieved from the hospital electronic medical record (EMR) system for patients presenting to the ED with acute traumatic injuries between January 2019 and April 2025. During this period, 10,722 trauma-related ED encounters were identified through an EMR export and constituted the sampling frame. From these encounters, ED encounters were randomly selected and assessed according to predefined eligibility criteria. Ineligible encounters were replaced by additional randomly selected encounters until 218 eligible ED encounters were included.

Study population and selection criteria

The study included patients aged 14 years and older who presented to the ED with a documented acute traumatic injury. This age threshold was based on the age classification used by the KKUH ED, where the Adult Emergency Unit manages patients aged 14 years and older, while the Pediatric Emergency Unit manages patients younger than 14 years. The observed age range was 14-91 years, with 91 years being the oldest patient during the study period.

Patients were eligible regardless of whether they were admitted or discharged after their initial ED evaluation. Injury mechanisms were classified according to the documented cause of injury, including traffic-related incidents, falls, burns, poisoning, sports trauma, lacerations, twisting injuries, and other traumatic mechanisms. Patients with non-traumatic conditions, those declared dead on arrival, or those with substantially incomplete medical records were excluded.

Data collection and outcomes

Data were extracted by a single researcher using a standardized data collection sheet and included age, sex, mechanism of injury, comorbidities, hospital admission, and LOS. The primary outcome was hospital admission following the initial ED evaluation. LOS among admitted patients was considered a secondary and exploratory outcome. The data abstractor was blinded to hospital admission status and LOS when assigning injury mechanisms and comorbidities. Hospital admission was defined as admission following the initial ED evaluation, while LOS was defined as the duration of hospitalization and reported as median (Q1, Q3).

Sample size justification and sampling

During the study period from January 2019 to April 2025, an EMR export identified 10,722 trauma-related ED encounters at KKUH. These 10,722 ED encounters constituted the sampling frame for the study. The sampling unit was the ED encounter rather than the unique patient; therefore, a patient could potentially contribute more than one encounter during the study period. Each encounter represented a separate ED visit.

The 10,722 encounters were compiled into a sampling list and randomly selected using a computer-generated random number sequence. Each selected encounter was reviewed against the predefined inclusion and exclusion criteria. When a selected encounter was found to be ineligible, it was replaced by another randomly selected encounter from the sampling frame. This process continued until 218 eligible ED encounters were identified for the final study sample.

Simple random sampling was therefore used to select ED encounters from the 10,722 trauma-related encounters available during the study period. Selected encounters were assessed sequentially against the predefined inclusion and exclusion criteria. Encounters that did not meet the eligibility criteria, including non-traumatic presentations, death on arrival, or substantially incomplete critical data, were excluded and replaced by additional randomly selected encounters. Because the number of replaced encounters was not prospectively aggregated by exclusion category, exact counts of all screened and excluded encounters could not be reliably reconstructed retrospectively.

A formal a priori sample size or statistical power calculation was not performed; the final sample size of 218 eligible ED encounters was determined pragmatically based on the feasibility and resources available for detailed retrospective chart review, while maintaining random selection from the available sampling frame. The adequacy of the achieved sample was subsequently assessed using a precision-based approach based on the standard formula using $n = \frac{Z^2 P(1-P)}{d^2}$ for estimating a single population proportion. Assuming the most conservative population proportion (P = 0.50), a 95% confidence level, and applying a finite population correction for the sampling frame of 10,722 encounters, the achieved sample of 218 encounters permits estimation of population proportions with a maximum margin of error of approximately ±6.6%.

Data extraction and standardized variables

Eligible cases were identified from the KKUH EMR system according to the predefined selection criteria. Relevant data were extracted by a single researcher and initially structured using a standardized digital collection sheet before being prepared for statistical analysis using Stata version 17.0 (StataCorp LLC, College Station, TX, USA; released 2021). The data abstractor was blinded to hospital admission status and LOS when assigning injury mechanisms and comorbidities. Double data abstraction and formal inter-rater agreement assessment were not performed; disagreement resolution was therefore not applicable.

Methodological limitations

A major limitation of this study is its reliance on retrospective electronic health records, which lacked consistent documentation of standardized physiological or anatomical trauma severity metrics. Specifically, data regarding the Injury Severity Score (ISS) and Glasgow Coma Scale (GCS), as well as formalized triage acuity scores, were unavailable or incomplete across the registry. Consequently, the analysis could not fully adjust for baseline injury severity, which represents a key confounding variable in predicting hospitalization and overall LOS.

Ethical considerations and confidentiality

This study was reviewed and approved by the Institutional Review Board (IRB) of King Saud University Medical City (KSUMC), King Saud University, Riyadh, Saudi Arabia (Project No. E-25-9981; approved 22 July 2025), following expedited review. Given the retrospective design and the exclusive use of de-identified records, the requirement for informed consent was waived by the IRB. The study was conducted in accordance with the Declaration of Helsinki, the International Council for Harmonization-Good Clinical Practice (ICH-GCP) guidelines, and the regulations of the Government of Saudi Arabia. To maintain patient confidentiality, all direct identifiers were removed, records were pseudonymized prior to statistical analysis, and access to the final dataset was restricted to the primary research team.

Statistical analysis

Data were analyzed using Stata. Continuous variables were non-normally distributed and were summarized as median (interquartile range (IQR)); group comparisons used the Mann-Whitney U test for two groups, and correlation with a second continuous variable used Spearman’s rank correlation. Categorical variables are summarized as frequency (percentage); group comparisons used the chi-square test where expected cell counts were adequate and Fisher’s exact test where cell counts were small.

The injury-mechanism items and comorbidity items were not mutually exclusive and are reported descriptively at full granularity. For inferential comparisons against admission status and LOS, individual mechanism items with fewer than five affected patients produce unstable estimates; these were therefore combined into five broader, non-mutually-exclusive mechanism categories (traffic-related (including motor-vehicle, motorcycle, and pedestrian sub-types), falls, burns/scalds, poisoning, and other trauma) and a single “any comorbidity” indicator for these comparisons.

For hospital admission, a multivariable logistic regression model was specified a priori and restricted to four predictors (age, sex, broad traffic-related mechanism, and any comorbidity) given the limited number of admissions. Both univariate (crude) and multivariable (adjusted) odds ratios are reported. LOS was available only for admitted patients who did not die on the day of admission (n=30 of 32 admitted). A two-sided P value of <0.05 was considered statistically significant throughout.

## Results

A total of 218 patients met the inclusion criteria, with a median age of 29.5 years (IQR 22-39; range 14-91); 145 (66.5%) were male (Table 1). Any comorbidity was present in 78 patients (35.8%), and 33 patients (15.1%) had ≥2 concurrent comorbidities, which is why the individual comorbidity percentages in Table 1 do not sum to the “Any comorbidity” total. The most frequent individual comorbidities were hypertension (11.0%), other unspecified comorbidities (9.6%), and diabetes mellitus (7.4% of 217 patients with data).

**Table 1:** Demographics and comorbidities of the studied patients (n=218) Values are n (%) unless stated otherwise. ^a^ n=217 (one missing value). Percentages for individual comorbidities do not sum to the “any comorbidity” total because patients could have more than one comorbidity.

Characteristic	Frequency (n)	Percentage (%)
Age (years), median (IQR)	29.5 (22-39)	
Sex: Male	145	66.5
Sex: Female	73	33.5
Comorbidities and prior conditions		
Diabetes mellitus^a^	16/217	7.4
Hypertension	24	11.0
Dyslipidemia	13	6.0
Cardiovascular condition	10	4.6
Osteoporosis	5	2.3
Hypothyroidism	10	4.6
Smoking history^a^	13/217	6.0
Renal disease	4	1.8
Epilepsy	11	5.0
Asthma	12	5.5
Other comorbidity (unspecified)	21	9.6
Any comorbidity	78	35.8
≥2 concurrent comorbidities	33	15.1

The predominant mechanisms of injury were falls (49.1%) and traffic-related incidents (22.0%), followed by unspecified injuries (12.8%), sports trauma (8.7%), and motor vehicle collisions (8.3%); the remaining mechanisms each affected fewer than 8% of patients (Table 2).

**Table 2:** Mechanisms of injury among the studied patients (n=218) Values are n (%). Categories are not mutually exclusive (patients could have more than one documented mechanism), so percentages sum to more than 100%.

Item	Frequency (n)	Percentage (%)
Falls	107	49.1
Traffic-related incidents	48	22.0
Sports trauma	19	8.7
Motor vehicle collisions	18	8.3
Twisting injury	17	7.8
Laceration	15	6.9
Closed door on finger	6	2.8
Poisoning	5	2.3
Pedestrian	5	2.3
Motorcycle collisions	4	1.8
Fight	4	1.8
Burns	3	1.4
Scalds	2	0.9
Pinprick injury	2	0.9
Unspecific injuries	28	12.8

Thirty-two patients (14.7%, 95% CI 10.3-20.1%) required hospital admission (Table 3). Among the 30 admitted patients with a recorded LOS (two patients died on the day of admission and were excluded from LOS analyses by definition), median LOS was 5 days (IQR 2-14; range 1-39).

**Table 3:** Admission and length of stay summary (n=218) ^a^ Among the 30 admitted patients with recorded LOS (range 1-39 days), two admitted patients died on the day of admission and have no LOS by definition.

Item	Frequency (n)	Percentage (%)	Other value
Not admitted	186	85.3	-
Admitted	32	14.7	95% CI (10.3-20.1)
Length of stay (LOS), days, median (IQR)^a^	-	-	5 (2-14)

Among comorbidities (Table 4), cardiovascular conditions (12.5% vs 3.2%, p=0.043), smoking history (22.6% vs 3.2%, p=0.001), and other unspecified comorbidities (21.9% vs 7.5%, p=0.020) were significantly more frequent among admitted patients. The overall “Any comorbidity” indicator was numerically higher among admitted patients (46.9% vs 33.9%), but the difference did not reach significance (p=0.156). Sex distribution and age did not differ significantly between admitted and non-admitted patients (p=0.271 and p=0.121, respectively), though median age was numerically higher among admitted patients (32.5 vs 28.5 years).

**Table 4:** Demographics and comorbidities by admission status Values are n (%) unless stated otherwise. P-values are from the chi-square test or Fisher's exact test for categorical variables and the Mann-Whitney U test for age. ^a^ Diabetes and smoking history denominators reflect one missing value each. * P<0.05 was considered statistically significant.

Characteristic	Not admitted (n=186)	Not admitted (%)	Admitted (n=32)	Admitted (%)	P-value
Sex: Male	121	65.1	24	75.0	0.271
Sex: Female	65	34.9	8	25.0	0.271
Age (years), median (IQR)	28.5 (22-38)	-	32.5 (23-44)	-	0.121
Diabetes^a^	13/185	7.0	3/32	9.4	0.712
Hypertension	20	10.8	4	12.5	0.761
Dyslipidemia	9	4.8	4	12.5	0.105
Cardiovascular condition	6	3.2	4	12.5	0.043*
Osteoporosis	4	2.2	1	3.1	0.551
Hypothyroidism	9	4.8	1	3.1	1.000
Smoking history^a^	6/186	3.2	7/31	22.6	0.001*
Renal disease	2	1.1	2	6.3	0.104
Epilepsy	8	4.3	3	9.4	0.208
Asthma	10	5.4	2	6.3	0.691
Other comorbidity	14	7.5	7	21.9	0.020*
Any comorbidity	63	33.9	15	46.9	0.156

Admission was significantly more common among patients with a broad traffic-related mechanism (43.8% of admitted vs 22.0% of non-admitted patients, p=0.009) and significantly less common among those with an “Other trauma” mechanism (18.8% vs 40.3%, p=0.020) (Table 5). Poisoning was strongly associated with admission (9.4% of admitted vs 1.1% of non-admitted patients, p=0.024), though this is based on only five total poisoning cases in the cohort. Falls and burns/scalds showed no significant association with admission (p=0.514 and p=0.473, respectively).

**Table 5:** Injury mechanism by admission status Values are n (%). Categories are not mutually exclusive; 33 patients (15.1%) had ≥2 broad mechanisms. ^a^ Chi-square or Fisher's exact test. * P<0.05 was considered statistically significant. MVC: motor vehicle collision

Mechanism	Not admitted (n=186)	Not admitted (%)	Admitted (n=32)	Admitted (%)	P-valueᵃ
Traffic-related (including MVC/motorcycle/pedestrian)	41	22.0	14	43.8	0.009*
Falls	93	50.0	14	43.8	0.514
Burns/scalds	3	1.6	1	3.1	0.473
Poisoning	2	1.1	3	9.4	0.024*
Other trauma	75	40.3	6	18.8	0.020*

LOS did not differ significantly by sex (male median 3.5 days (IQR 2-15) vs female 7.5 days (IQR 4-9.5), p=0.540) or by presence of any comorbidity (p=0.214), although LOS was numerically longer among patients with any comorbidity (median 9 days, IQR 2-15, n=13) than without (median 3 days, IQR 2-7, n=17) (Table 6). Age was not correlated with LOS (Spearman's rho=-0.115, p=0.544, n=30).

**Table 6:** Length of stay (LOS) by demographics and comorbidity status among admitted patients (n=30) ^a^ Mann-Whitney U test; Spearman’s rank correlation was used for age. IQR: interquartile range

Characteristic	n	Median LOS, days (IQR)	P-value
Sex: Male	22	3.5 (2-15)	0.540^a^
Sex: Female	8	7.5 (4-9.5)	0.540^a^
Any comorbidity: No	17	3 (2-7)	0.214^a^
Any comorbidity: Yes	13	9 (2-15)	0.214^a^
Age	30	Spearman's rho = -0.115	0.544

By injury mechanism, LOS was significantly longer among the three patients with poisoning (median 15 days, IQR 10-31) than the 27 without (median 4 days, IQR 2-9; exact p=0.042). LOS did not differ significantly by broad traffic-related mechanism (median 6.5 vs 3.5 days, p=0.598) or by falls (median 3 vs 8 days, p=0.147).

In the pre-specified multivariable logistic regression model (Table 7; n=218, 32 events), broad traffic-related mechanism was associated with higher odds of hospital admission after adjustment for age, sex, and any comorbidity (adjusted OR 3.41, 95% CI 1.49-7.83, p=0.004).

**Table 7:** Multivariable logistic regression for hospital admission (n=218) Adjusted model: n=218, with 32 admission events; likelihood ratio (LR) χ^2^(4)=13.68, P=0.008, pseudo-R^2^=0.075. Hosmer-Lemeshow goodness-of-fit χ^2^(8)=4.96, P=0.762. Area under the receiver operating characteristic curve (AUC)=0.69. *P<0.05 was considered statistically significant.

Predictor	Crude OR	Crude 95% CI	Crude p-value	Adjusted OR	Adjusted 95% CI	Adjusted p-value
Age (years)	1.02	1.00, 1.04	0.078	1.02	1.00, 1.05	0.069
Sex (female vs male)	0.62	0.25, 1.41	0.274	0.53	0.22, 1.28	0.158
Traffic-related mechanism	2.75	1.25, 5.99	0.011*	3.41	1.49, 7.83	0.004*
Any comorbidity	1.72	0.80, 3.68	0.160	1.45	0.60, 3.47	0.411

Age (crude OR 1.02, p=0.078; adjusted OR 1.02, p=0.069), sex (crude OR 0.62, p=0.274; adjusted OR 0.53, p=0.158), and any comorbidity (crude OR 1.72, p=0.160; adjusted OR 1.45, p=0.411) were not significantly associated with admission in either univariate or multivariable analysis.

## Discussion

This study examined the association between injury mechanisms, hospital admission, and LOS among trauma patients presenting to the ED in Riyadh, Saudi Arabia. Three findings are particularly important. First, falls were the most frequently documented mechanism of injury, accounting for nearly half of the cohort, whereas traffic-related injuries were less frequent. Second, despite their lower frequency, traffic-related injuries were associated with substantially higher odds of hospital admission after adjustment for age, sex, and any comorbidity. Third, poisoning was associated with longer hospital stay among admitted patients, although this finding was based on very few cases and should therefore be interpreted cautiously. Together, these findings demonstrate an important distinction between mechanisms that account for a large volume of ED presentations and those that are more strongly associated with subsequent hospital utilization.

Falls accounted for 49.1% of patients in the present study, whereas traffic-related incidents accounted for 22.0%. This distribution differs substantially from previous Saudi trauma data. Alghnam et al., using a hospital-based trauma registry from a large trauma center in Riyadh, reported traffic-related injuries as the most frequent mechanism (52.0%), followed by falls (23.4%) [9]. In contrast, falls have been reported as the most common cause of traumatic injury in large inpatient datasets from other high-income settings [10]. The predominance of falls in our cohort therefore requires consideration of how trauma cases were identified rather than simply comparison of percentages across studies.

Several methodological and healthcare setting factors may explain this difference. The present study included trauma patients presenting to the ED across a broad spectrum of injury severity, including the large majority who were not subsequently admitted. In contrast, the Saudi study by Alghnam et al. was based on a trauma registry from a large trauma center [9], a setting more likely to capture clinically significant injuries than a broad ED-based sample. Consequently, lower-acuity injuries, including falls that can be evaluated and discharged directly from the ED, may be more visible in our study, whereas high-energy traffic injuries may be disproportionately represented in trauma-center registries. This interpretation is consistent with our own data: although falls constituted nearly half of all presentations, their frequency did not differ significantly between admitted and non-admitted patients. Differences in institutional case mix, catchment populations, ambulance destination, and referral pathways could also contribute to differences between centers. However, these factors were not directly measured in the present study and should be regarded as plausible explanations rather than established causes of the observed difference. Finally, the single-center sampling strategy may itself have influenced the observed mechanism distribution. Therefore, the predominance of falls in this cohort should be interpreted as a characteristic of the sampled ED population and should not be extrapolated to imply that falls have replaced road traffic injuries as the predominant mechanism of trauma in Riyadh or Saudi Arabia.

The relationship between injury frequency and admission further illustrates this distinction. Falls were common but were not significantly associated with admission, whereas traffic-related injuries were substantially more frequent among admitted patients and remained associated with admission after adjustment for age, sex, and any comorbidity (adjusted OR 3.41, 95% CI 1.49-7.83).

This suggests that the mechanism responsible for the greatest number of ED presentations is not necessarily the mechanism associated with the greatest need for inpatient care. A plausible explanation is the greater energy transfer associated with road traffic collisions and their potential to produce fractures, internal injuries, and multisystem trauma requiring observation, operative treatment, or critical care. Saudi data support the substantial clinical burden associated with road traffic trauma. For example, a hospital-based study of patients presenting with road traffic accidents in the Eastern Province reported requirements for early surgical intervention and surgical intensive care admission among a subset of patients [15]. Thus, our findings complement rather than necessarily contradict trauma-registry studies in which traffic injuries predominate: falls may contribute substantially to the overall volume of ED trauma, while traffic-related mechanisms may account for a disproportionate share of patients requiring hospitalization.

Comorbidity showed a more nuanced relationship with admission. Cardiovascular conditions, smoking history, and other unspecified comorbidities were individually more frequent among admitted patients in unadjusted comparisons. However, the broader indicator of having any comorbidity was not significantly associated with admission and remained non-significant after multivariable adjustment. This is important because the unadjusted associations should not be interpreted as evidence that comorbidity independently predicts admission in this cohort. Previous evidence from major-trauma populations has linked pre-existing comorbidity, particularly cardiovascular, metabolic, hepatic, and renal disease, with increased morbidity, complications, LOS, and mortality [12]. The absence of an independent association in our study may partly reflect differences in case mix: our cohort included a broad ED trauma population rather than exclusively major trauma, had a relatively young age distribution, and contained only 32 admission events. Furthermore, combining heterogeneous conditions into a single “any comorbidity” variable may attenuate associations that are specific to particular diseases or levels of comorbidity burden. These factors, together with the limited number of events, may have reduced the ability to identify modest independent effects.

Neither age nor sex was significantly associated with admission after adjustment. Admitted patients were numerically older than non-admitted patients, but the difference was not statistically significant, and age did not independently predict admission in the final model. This should not be interpreted as evidence that age is unimportant in trauma outcomes generally. Rather, the relatively young distribution of the present cohort, the inclusion of both lower- and higher-acuity ED trauma, and the limited number of admissions may have reduced the ability to detect an age-related effect. More importantly, standardized measures of injury severity were unavailable, making it difficult to separate the influence of patient characteristics from the severity of the traumatic event itself.

Among admitted patients with available length-of-stay data, the median hospital stay was five days. LOS did not differ significantly according to sex, age, the presence of any comorbidity, traffic-related injury, or falls. Although patients with any comorbidity had a numerically longer median stay than those without comorbidity, this difference did not reach statistical significance. The absence of significant associations should be interpreted cautiously because LOS analyses were restricted to only 30 patients after excluding two patients who died on the day of admission, a restriction that may affect the interpretation and generalizability of the LOS findings. Accordingly, these analyses had limited statistical power, particularly for less frequent injury mechanisms. In addition, LOS is influenced by factors beyond injury mechanism, including injury severity, complications, need for surgery, rehabilitation requirements, and institutional discharge practices, several of which could not be evaluated in the available data.

Poisoning was the only mechanism significantly associated with longer LOS, with a median stay of 15 days among the three admitted patients with poisoning compared with four days among patients without poisoning. Previous research has shown that hospitalization following poisoning can vary substantially according to the nature and circumstances of exposure; intentional and potentially hazardous poisoning have been associated with prolonged hospitalization [16]. Nevertheless, the present result is based on only three admitted poisoning cases. A single prolonged admission could therefore have a substantial influence on the estimated median and statistical comparison. The association should consequently be regarded as exploratory and hypothesis-generating rather than evidence that poisoning consistently causes longer hospitalization in this population.

Taken together, our findings emphasize that frequency of presentation and intensity of healthcare utilization are related but distinct dimensions of trauma burden. Falls accounted for the largest proportion of trauma presentations in this ED-based cohort, but traffic-related injuries were more strongly associated with hospital admission. This distinction has practical implications for trauma service planning. EDs may need to accommodate a substantial volume of fall-related presentations, while traffic-related trauma may require greater readiness for inpatient evaluation and management. At the same time, because injury severity and institutional referral patterns were not measured, these findings should not be used to infer differences in intrinsic severity between mechanisms or to estimate the population-wide distribution of trauma in Saudi Arabia.

These findings also have implications for trauma prevention strategies. The high frequency of falls suggests that fall-prevention measures, including environmental safety interventions, patient and caregiver education, and targeted assessment of modifiable fall risks, may help reduce the substantial volume of fall-related ED presentations. In parallel, the stronger association between traffic-related injuries and hospital admission supports continued emphasis on road-safety measures, including enforcement of traffic regulations, injury-prevention education, and interventions targeting high-risk road-user behaviors. From a trauma-care perspective, these findings may help inform resource allocation by recognizing that mechanisms generating the highest ED volume are not necessarily those associated with the greatest inpatient utilization. However, the absence of standardized injury-severity measures means that the observed association between traffic-related injuries and admission should be interpreted cautiously, as residual confounding by injury severity cannot be excluded.

The present findings should also be interpreted in light of several limitations. The retrospective design relies on the completeness and accuracy of EMRs and is therefore susceptible to information, coding, and classification bias. Although the data abstractor was blinded to hospital admission status and LOS when assigning injury mechanisms and comorbidities, all data were abstracted by a single researcher without independent second abstraction or formal inter-rater agreement assessment. The presence of overlapping and occasionally ambiguous mechanism categories may therefore have introduced misclassification or classification bias. Injury-mechanism categories were not mutually exclusive, and some individual mechanisms occurred infrequently. In addition, this was a single-center study, and the distribution of trauma mechanisms may reflect the specific case mix, urban catchment, referral environment, and patterns of emergency care at the study institution rather than the broader Riyadh or Saudi Arabian trauma population. Potential selection effects may also have influenced the final sample, as ineligible or substantially incomplete records were replaced during sampling. Referral pathways and ambulance destination patterns were not directly measured, and therefore their potential contribution to the observed mechanism distribution remains speculative. Most importantly, standardized indicators of injury severity, including the ISS [17], GCS [18], and formal triage acuity measures, were unavailable or inconsistently documented. Residual confounding by injury severity is therefore an important limitation, particularly when interpreting the association between traffic-related injuries and admission. Accordingly, the observed association between traffic-related injury and hospital admission should not be interpreted as evidence of an independent effect of injury mechanism, because differences in underlying injury severity could not be adequately accounted for. The limited number of admission events also restricts the precision of the multivariable estimates, while the LOS analysis included only 30 patients. In particular, the association between poisoning and longer LOS was based on only three admitted patients and should therefore be regarded as exploratory and hypothesis-generating rather than confirmatory. In addition, multiple statistical comparisons were performed, increasing the possibility of chance findings; therefore, the reported associations should be interpreted cautiously and require confirmation in larger studies.

These limitations underscore the need for larger multicenter studies using standardized injury-severity measures, consistent trauma coding, and more detailed information on referral pathways to determine whether these findings persist across different trauma-care settings in Saudi Arabia.

## Conclusions

In this single-center cohort of trauma patients presenting to the ED, falls were the most frequently documented injury mechanism, whereas traffic-related injuries were associated with higher odds of hospital admission after adjustment for age, sex, and any comorbidity. Poisoning was associated with longer hospital stay; however, this finding was based on only three admitted patients and should therefore be regarded as exploratory and hypothesis-generating rather than confirmatory. These findings highlight that the mechanisms contributing most frequently to ED trauma presentations may differ from those associated with greater inpatient utilization. However, because standardized measures of injury severity were unavailable or inconsistently documented, residual confounding cannot be excluded. Larger multicenter studies incorporating standardized measures of injury severity are needed to determine whether these findings are consistent across trauma-care settings in Saudi Arabia.

## References

[1] Langenberger B, Worsham C, Geldsetzer P (2024). The effect of length of stay in hospital on patients’ health outcomes: a quasi-experimental study [PREPRINT]. medRxiv.

[2] Alharbi RJ, Lewis V, Mosley I, Miller C (2020). Current trauma care system in Saudi Arabia: a scoping literature review. Accid Anal Prev.

[3] Alghnam S, Towhari JA, Al Babtain I, Al Nahdi M, Aldebasi MH, Alyami M, Alkhalaf H (2019). The associations between injury mechanism and extended hospital stay among pediatric patients: findings from a trauma Center in Saudi Arabia. BMC Pediatr.

[4] Taylor SL, Sen S, Greenhalgh DG, Lawless M, Curri T, Palmieri TL (2015). A competing risk analysis for hospital length of stay in patients with burns. JAMA Surg.

[5] Pytlak MA, Tomska N, Bohatyrewicz A, Rył A, Rotter I (2016). Influence of selected demographic factors on traumas in persons over 65 years of age reporting to the Hospital Medical Ward. Fam Med Prim Care Rev.

[6] Haghparast-Bidgoli H, Saadat S, Bogg L, Yarmohammadian MH, Hasselberg M (2013). Factors affecting hospital length of stay and hospital charges associated with road traffic-related injuries in Iran. BMC Health Serv Res.

[7] Doctoroff L, Hsu DJ, Mukamal KJ (2017). Trends in prolonged hospitalizations in the United States from 2001 to 2012: a longitudinal cohort study. Am J Med.

[8] Albabtain IT, Alfehaid O, Alghunaim M (2019). Epidemiology of adult trauma at a tertiary hospital in Riyadh, Saudi Arabia. Int J Med Res Health Sci.

[9] Alghnam S, Alkelya M, Al-Bedah K, Al-Enazi S (2014). Burden of traumatic injuries in Saudi Arabia: lessons from a major trauma registry in Riyadh, Saudi Arabia. Ann Saudi Med.

[10] DiMaggio C, Ayoung-Chee P, Shinseki M (2016). Traumatic injury in the United States: in-patient epidemiology 2000-2011. Injury.

[11] Yıldız GÖ, Sertçakacılar G, Tontu F, Akyol D (2022). The effect of comorbidities and obesity on postoperative outcomes. Med J Bakirkoy.

[12] Glynn R, Edwards F, Wullschleger M, Gardiner B, Laupland KB (2025). Major trauma and comorbidity: a scoping review. Eur J Trauma Emerg Surg.

[13] Samoborec S, Simpson P, Hassani-Mahmooei B, Ruseckaite R, Giummarra M, Ayton D, Evans S (2020). Impact of comorbidity on health outcome after a transport-related injury. Inj Prev.

[14] Glance LG, Stone PW, Mukamel DB, Dick AW (2011). Increases in mortality, length of stay, and cost associated with hospital-acquired infections in trauma patients. Arch Surg.

[15] Amir A, Amir B, Alghannam A (2023). Analysis of road traffic injuries presented to the emergency department in the Eastern Province of Saudi Arabia: a hospital-based study. J Med Life.

[16] Thomas SH, Lewis S, Bevan L (1996). Factors affecting hospital admission and length of stay of poisoned patients in the north east of England. Hum Exp Toxicol.

[17] Baker SP, O’Neill B, Haddon W Jr, Long WB (1974). The injury severity score: a method for describing patients with multiple injuries and evaluating emergency care. J Trauma.

[18] Teasdale G, Jennett B (1974). Assessment of coma and impaired consciousness. A practical scale. Lancet.

